# Correlations between *Lumbricus terrestris* survival and gut microbiota

**DOI:** 10.3402/mehd.v23i0.17316

**Published:** 2012-04-24

**Authors:** Knut Rudi, Knut Olav Strætkvern

**Affiliations:** 1 Department of Natural Sciences and Technology, Hedmark University College, Hamar, Norway; 2 Department of Chemistry, Biotechnology and Food Science, Norwegian University of Life Sciences, Ås, Norway

**Keywords:** earthworm, 16S rRNA gene, gut, cellulose

## Abstract

**Background:**

The interplay between diet, gut bacteria and health still remain enigmatic. Here, we addressed this issue through the investigation of the effect of crystalline cellulose on the earthworm *Lumbricus terrestris* gut microbiota composition and survival.

**Methods:**

Earthworm gut contents were analyzed after 14 days of feeding using a mixed 16S rRNA gene sequencing approach, in addition to direct measurements of cellulase activity. The survival of earthworms was followed each week for 17 weeks.

**Results:**

We found a tendency that the crystalline cellulose fed earthworms survived better than the high energy fed earthworms (*p*=0.08). Independent of feeding we found that the bacterial group related to *Ferrimonadaceae* was correlated to an increased lifespan (*p*=0.01). We also found a positive correlation between *Ruminococcaceae* related bacteria and cellulase activity in the earthworm gut (*p*=0.05). Surprisingly, however, the cellulase activity was not correlated to the feeding regime.

**Conclusion:**

Taken together, the interactions between diet, gut microbiota and lifespan seem complex.

The interaction between the host and its gut microbiota is of fundamental importance for host health and disease (1). Vertebrates, invertebrates (including earthworms) utilize the gut bacteria for provision of metabolic capacities and protection against pathogens (2). There have been numerous studies on the earthworm gut microbiota (3–6). Although several studies have suggested earthworm associated bacteria, none of the previous studies, however, have addressed the question of whether there is a correlation between diet, gut microbiota, and earthworm survival.

Human activities have dramatically altered the nutrient status of soil, and thereby soil micro- and macro-organisms. *Lumbricus terrestris* is an anecic surface feeding earthworm that is particularly vulnerable to environmental conditions such as nutrient and microbial status (7, 8). Understanding of the factors contributing to the survival of this earthworm would therefore be important due to the large environmental impact of *L. terrestris*.

The aim of the present work was to evaluate the effect of feed and gut microbiota with respect to *L. terrestris* survival. This was done in a controlled feeding experiment – testing the effect of crystalline cellulose. The gut the gut microbiota was quantified by a mixed sequencing approach, in addition to measurements of cellulase activity and survival.

We chose an artificial soil microecosystem in our evaluations, since it is very difficult to control the environmental conditions in natural ecosystems. For earthworms there is an ongoing debate of whether the main cellolytic activity in the gut is host or microbial derived (9). This is the rationale for the choice to evaluate the effect of crystalline cellulose in our feeding experiments. The choice of using a complete microbiota screening approach was based on the fact that the gut microbiota in earthworms is not yet well-described.

## Materials and methods

### Experimental set-up

Six control earthworms were given 1 g Magic worm food (Magic products, Wisconsin, USA) weekly, while the other six earthworms were given 1 g of 1:1 mix of Magic worm food and crystalline cellulose (Sigma, St. Louis, Missouri). Magic worm food is a corn-based feed contains a combination of 32 different proteins, fats, minerals, vitamins, and carbohydrates which are essential to the earthworm (www.magicproducts.com).

The gut content of the earthworms were analyzed after 14 days of feeding. This was done by cutting an approximately 1 cm piece of the anterior part of live earthworm. The gut content was subsequently squeezed out, and immediately frozen at –80°C.

### Measurements of cellulase activity and protein concentration

Cellulase was assayed in black microtiter plates (NUNC F96) using fluorescent substrate resorufin cellobioside (Marker Gene Technologies Inc., Eugene, Oregon, USA), specific for the β-glucosidase activity of cellulases. Extracts of homogenized earthworm gut content were prepared directly in the assay reaction buffer (0.1M sodium acetate, pH 6.0). Undiluted extracts (50 µl) were analyzed in duplicate wells by adding 50 µl of 0.5 mM substrate, and incubating at 25°C for approx. 10 min. Filter settings for detection of released resorufin were emission 575 nm and excitation 545 nm. Employing kinetics mode of the plate reader (FluoStar Optima, BMG Labtech GmbH, Offenburg, Germany), the cellulase activity was calculated from the slope of curves (relative fluorescence/min). *Trichoderma reesei* cellulase, diluted to 0.084 units/ml (Sigma C2730) served as positive control.

Soluble protein (µg/ml) in extracts was determined with the Quant-iT protein assay (Invitrogen, Carlsbad, California, USA) and used to obtain specific enzyme activity (units/mg protein).

### DNA extraction

Gut content (400 mg) was dissolved in TE buffer in a total volume of 500 µl. Into each of 2.0 ml tubes with screw caps and silica rings in the lids glass beads were added. 90 µl of each sample were transferred to tubes with glass beads (250 mg, 106 microns and finer, Sigma-Aldrich, Steinheim, Germany) and 1200 µl binding buffer. Bacteria cells were mechanically lysed in the Bead Beater (1×5 min). All samples were centrifuged at 13,000 rpm for 5 min at RT and the supernatants (500 µl) were transfers to new microcentrifuge tubes containing 10 µl SiMAG/MP-DNA Magnetic Beads (4 mg/ml, Chemicell, Berlin, Germany). The tubes were then vortexed, incubated for 5 min at RT and centrifuged briefly. The tubes were placed on a magnetic separator for 30 second to remove and discard the supernatants. Washing Buffer I (1 ml) was added to each tube with gentle vortex at RT. The same washing steps were done with Washing Buffer II (1 ml, 5 seconds vortex) and Washing Buffer III (1 ml, only 1 second vortex). To elute DNA from the magnetic beads, 100 µl elution buffer was added to the tubes and incubated for 10 min at 60°C.

### PCR reaction

PCR amplification of the 16S rRNA genes was carried out using the primers (5′-TCC TAC GGG AGG CAG CAG T-3′) and (5′-GGA CTA CCA GGG TAT CTA ATC CTG TT-3′) (Nadkarni et al, 2002). The 25-µl PCR mixture contained 1× Hot Start buffer II (Finnzymes), 400 µM of a dNTPs mixture, DyNAzyme II Hot Start DNA polymerase (Finnzymes), 0.4 µM of each primer and 2 µl of the extracted DNA. The amplification profile was an initial step of 94°C for 10 min and then 30 cycles of 94°C for 30 seconds, 60°C for30 seconds, and 72°C for 30 seconds, and a final extension at 72°C for 7 min.

### Mixed sequencing

For each sample the following components were mixed: 1× seq buffer, 20 u/µl Exo I, dH_2_O, and PCR product to the total volume 5 µl. The samples were then incubated at 37°C for 60 min, 85°C for 15 min and ≤10°C at the end.

The 10 µl content of each tube was: Exo I treated PCR reaction sample, 0.25×sequencing buffer, Big Dye v.1.1 (Applied Biosystems, Foster City, CA), dH_2_O and the universally conserved primer U515FFc30 (0.32 pmol). The thermo-cycling steps were 96°C for 1 min and then 25 cycles of 95°C for 15 seconds, 50°C for 5 seconds, and 60°C for 4 min.

The final purification step for each sample was performed in the 60-µl mixture comprising of the product from sequencing PCR, 10 µl SAM and 45 µl exterminator reagents (Applied Biosystems). The sample were vortexed at 1,500 rpm for 30 min, with subsequent centrifugation at 2500 rpm for 2 min.

### Bioinformatic and statistical analyses

The mixed DNA sequence spectra were resolved using Multivariate Curve Resolution (MCR) as previously described (10). The MCR spectra were subsequently base-called using an in-house developed base-caller. The identity of the base-called sequences was determined using the Classifier tool in the RDP database (http://rdp.cme.msu.edu).

All statistical analyses in this work were done using the statistical software SYSSTAT 13. The survival analyses were conducted using non-parametric tests. The significance of the differences between the groups was determined by the Breslow-Gehan test. The underlying assumption for this test is that one group is at higher risk than the other. The overall relatedness of the gut microbiota between the earthworms in the experiment was determined by hierarchical centroide clustering with Euclidian distances. These analyses results in dendrograms reflecting hierarchical relatedness. Correlations between the microbiota and other factors were determined by partial least square regression. These analyses correct for correlations between the variables tested.

## Results

### Survival analyses

**Fig. 1:**
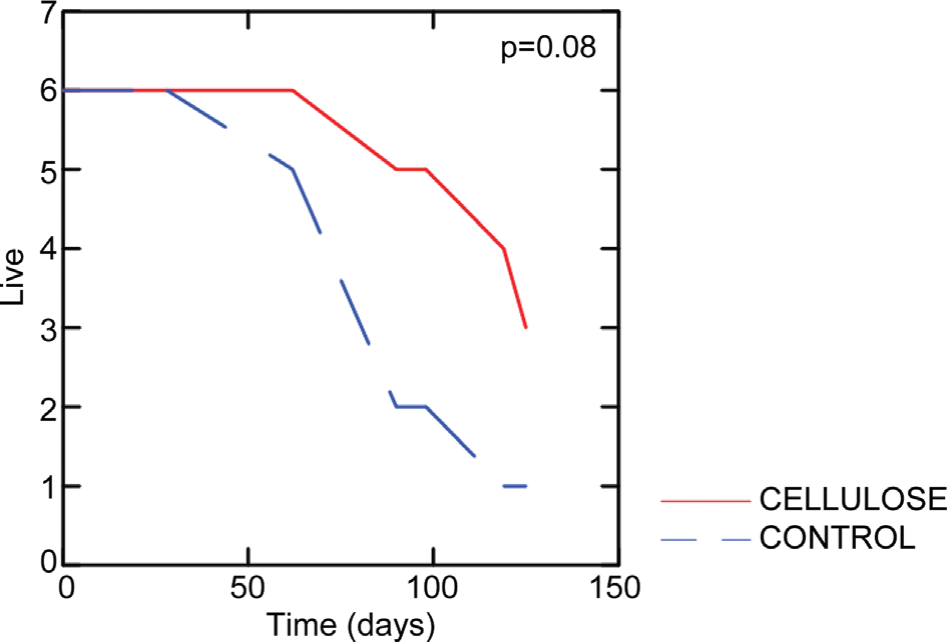
Earthworm survival curves. The color of the curves indicate feeding regime. The significance of the difference between the groups was determined by the Breslow-Gehan test.

The average survival time was approximately 100 days. For the earthworms given control feed the earthworms survived about 50 days before they started dying, while the crystalline cellulose feed earthworms started dying at about 70 days. Although not statistically significant (*p*=0.08), the results from the survival experiments indicated that the earthworms given crystalline cellulose survived better than the control earthworms (Fig. 1).

### Composition of the dominant microbiota

The composition of the earthworm gut microbiota was determined after 14 days of the experiment. This was done by MCR analysis, resulting in 5 components (Table 1). Classification of the main MCR resolved components by the RDP II classifier showed relatively poor classification support (Table 2). This was also confirmed by BLAST searches, where the hits with the lowest e-values were for uncultured bacteria. Interestingly, however, by Blast searches for the component classified as *Lachnospiraceae Incertae Sedis* (*IS*) actually showed 96% identity to *Candidatus Lumbricincola*, which is a deep-branching bacterium widespread among earthworms (11). This lineage was probably not included in the model for the RDP Classifier.

**Table 1:** DNA sequence of the MCR components

Name	DNA sequence (5′-3′)
Flavobacteriaceae	GCAAGCGTTATCCGGATTTATCTGGGTGTAAAGGGAGCSTAGARGGTTTGTTCAAGTCATGATGTGAYTCTCTCTGCGGCTCACCTGTGGAAACTGCATGTTGATACTGTGATTAGTCTTGAGTGRAGTWGGAGGAGGCGATCGTGAGTGMTAGGCGTTGGAATTGCGCGTATAGGAATATATTCAGCGAGAGACACTCAGATTGTGGCGAGGCGCTCACTAGAAGCTAGTA
Ruminococcaceae	CGAGCGTTATCCGGATTATCTGGCGTAAAGGCCGTAGCGTTAGTAGTCTAGTGTGAATCCAGTGGCTCAACCTTGGACTGCATTGATACTGCTGAGAACTATGAGTTCATGTGAGAGGTAGACGTGGATTCCGAMTGAGTGTAGCGGTGAATGCGTAGAGTATTTCASGAGARACCAGATGGCGAAGCGCGAGCTCTCATCTGCATCAAGTGATGA
Lachnospiraceae	AAGCGTTATCCGATTATCTGGGCGTAAAGCSATGTAGGCGTTTACTAAGTCTGGTGTGAAAGCTCACGGCCTAACCGTGAATTGCATTGGAAACTGGTGAACTAGAATACTGGAGGGGTAAGTGGAATTCCATGTGTAGCGGTGGAATGCGTAGATATATGGAGGAACACCAATGGCGAAGCGCAGCTTACTGGACAGAGA
Ferrimonadaceae	AGCGTTAGATTCGGATTACTGGGCGTAAAGCGCACSAGCGTTTGGWTAAGTTCAGATGTGAAATGCCCCGGGCTCAACCTGTGGARTGCATTTAGAATACTGTCCAGCTATGGAGTCTTGTYGAGGGGGGTAGAATTCTCAGGTGTAGCGGTGATGCGTAGAGATCTGAGAATCACCGGTGTGCGAAGGCGGCCCCCTGACAAATGA
Oceanospirillaceae	GCGAGSGGGTGAAGGGCCTGTGTATATCTGGCGTAAAACGCGCCCTCTAGCGGTTTTATTAAGTCTGGGTGAAATCCGGGCCTCAACCTTGGGACTGCTTTGAAACTGGGGCTGAGTTCGGGAGYGGAAGTGGATTCCTGTGTAGCGGTGAAATTGCGTAGATATTCTGGAGGACACCAGTGGCGAAGCGGCTCTACTGCCGTAC

**Table 2:** Classification of MCR components by the RDP hierarchical classifier^a^

MCR components^b^	Domain	Phylum	Class	Order	Family
Flavobacteriaceae is	**Bacteria**	Bacteroidetes	Flavobacteria	Flavobacteriales	Flavobacteriaceae
Ruminococcaceae is	**Bacteria**	Firmicutes	Clostridia	Clostridiales	Ruminococcaceae
Lachnospiraceae is	**Bacteria**	**Firmicutes**	Clostridia	Clostridiales	Lachnospiraceae
Ferrimonadaceae is	**Bacteria**	**Proteobacteria**	γ**-proteobacteria**	Alteromonadales	Ferrimonadaceae
Oceanospirillaceae is	**Bacteria**	Proteobacteria	γ-proteobacteria	Oceanospirillales	Oceanospirillaceae

a Bold indicate the bootstrap classification support >50%.

b The naming of the MCR components is based on the family names. Since none have bootstrap support >50% the classification is *incertae sedis(is)*.

**Fig. 2:**
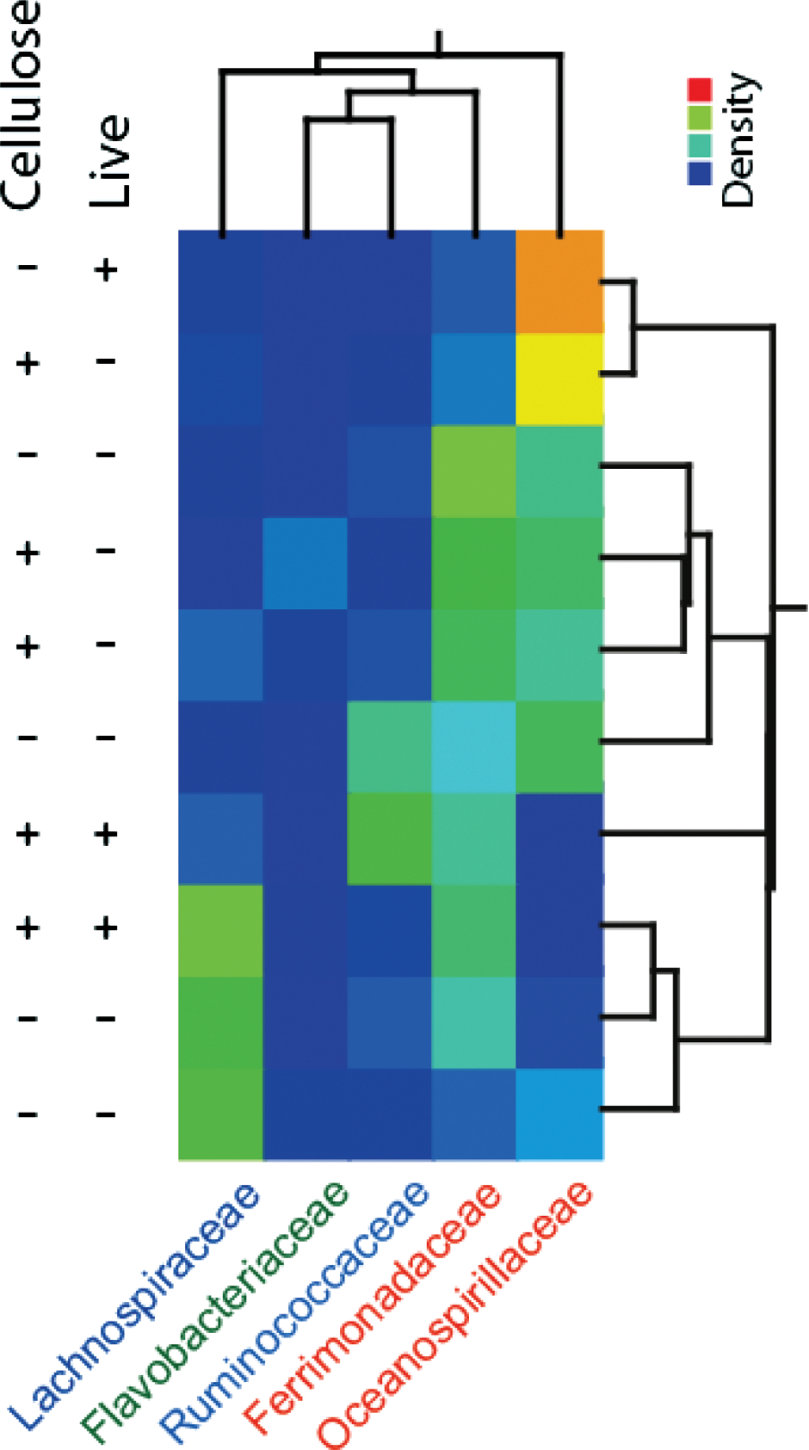
Gut microbiota clustering based on the concentration profiles for the MCR components. The concentration of each of the components in each sample is indicated by the color code. The clustering represents the relatedness based on the centroide linkage.

There were relatively large differences in the microbiota composition between the earthworms analyzed (Fig. 2). The component classified as *Ferrimonadaceae IS*, however, showed the most even distribution among the earthworms, while the other components showed relatively distinct distributions.

### Correlations of gut microbiota to viability, cellulase activity and feeding regime

**Fig. 3:**
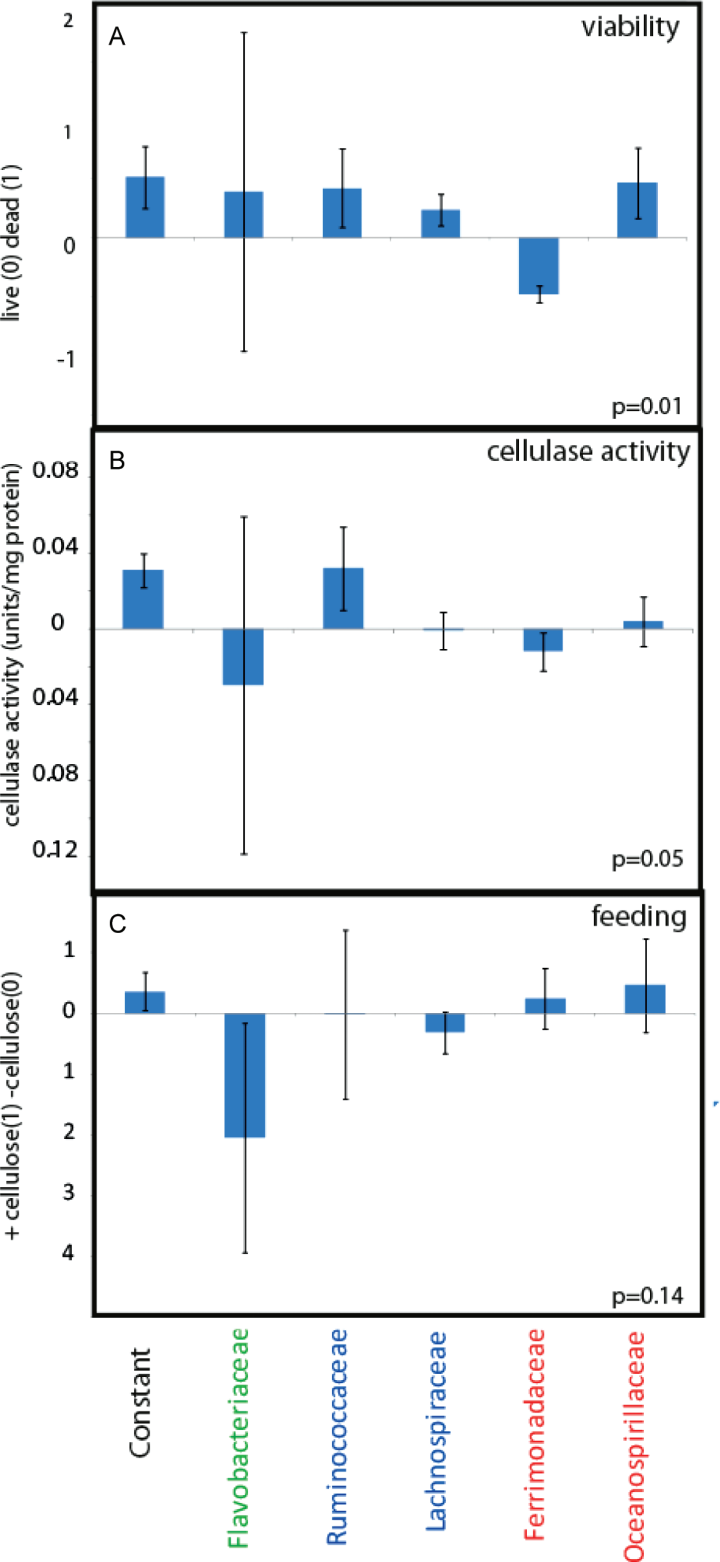
PLS regressions with the gut microbiota as predictors. (A) Live/dead correlations, (B) correlations to cellulase activity, and (C) correlations to feeding regime. The regression coefficients with standard deviations are shown.

The microbiota composition was significantly correlated to viability (*p*=0.01). The most pronounced composition was that *Ferromadaceae IS* was correlated to increased viability, while all the other bacterial groups seemed correlated with decreased viability (Fig. 3A).

There was also a significant correlation between cellulase activity and the gut microbiota (*p*=0.05). *Ruminococcaceae* *IS* was correlated to cellulase activity (Fig. 3B). Strangely, however, there was no or low correlations of *Ruminococcaceae IS* to the feeding regime (Fig. 3C). There were neither any correlations between feeding regime and cellulase activity (results not shown).

## Discussion

The most consistent finding was that *Ferrimonadaceae IS* was correlated to increased survival of *L. terrestris*. This bacterium also showed the most widespread distribution among the earthworms, suggesting potential symbiotic relations. This family relies strictly on respiration, with a range of electron acceptors, including iron (12). It would be highly interesting in future experimentation to determine if the increased survival can be explained by the maintenance of iron homeostasis through the reduction of Fe^3 +^ to the biologically active Fe^2 +^.

Bacteria belonging to *Ruminococcacea* are commonly associated with cellolytic activity. It is particularly interesting that these bacteria are common colonizers of the mammalian gut (13). Thus, in the future it would be interesting to determine the potential coevolution of cellulytic bacteria in mammalians and earthworms. The lack of correlation to crystalline cellulose feeding, however, was intriguing. It could be that the crystalline cellulose actually was inaccessible to degradation, and that it was relatively inert in the earthworm gut.

Interestingly, bacteria related to the *Candidatus Lumbricincola* (classified as *Lachnospiraceae IS* by RDP) dominated the gut microbiota in several of the earthworms. This bacterium is probably strictly associated with earthworms, since it has independently been identified in earthworms and no other environment (11). Furthermore, the high level in the gut lumen content suggests that there are indeed specific gut bacteria in earthworms. The function, however, still remains known, since no representatives have yet been cultured. It will be highly interesting in the future to determine the function of this bacterium in the earthworm gut.

In conclusion in this work we have shown several potentially interesting interactions between the gut microbiota and the earthworm host with respect to survival. These interactions will be highly interesting to follow up in future experiments.
